# rs1888747 polymorphism in the *FRMD3* gene, gene and protein expression: role in diabetic kidney disease

**DOI:** 10.1186/s13098-015-0121-5

**Published:** 2016-01-08

**Authors:** Marjoriê P. Buffon, Mariana P. Carpena, Denise A. Sortica, Andressa Santer, Rodrigo Carlessi, Bianca M. de Souza, Maria I. Edelweiss, Milton Berger, Daisy Crispim, Luís H. Canani

**Affiliations:** Endocrine Division, Hospital de Clínicas de Porto Alegre, Rua Ramiro Barcelos 2350, prédio 12, 4° andar, Porto Alegre, RS 90035-003 Brazil; Pathology Service, Hospital de Clínicas de Porto Alegre, Porto Alegre, RS Brazil; Urology Service, Hospital de Clínicas de Porto Alegre, Porto Alegre, RS Brazil; Post-Graduation Program in Medical Sciences: Endocrinology, Universidade Federal do Rio Grande do Sul, Porto Alegre, RS Brazil; Post-Graduation Program in Medical Sciences: Ginecology, Universidade Federal do Rio Grande do Sul, Porto Alegre, RS Brazil

**Keywords:** *FRMD3* gene expression, Polymorphism, Human kidney, Diabetic kidney disease

## Abstract

**Background:**

We carried out a case–control study in patients with type 2 diabetes mellitus (T2DM) to evaluate the association between seven single nucleotide polymorphisms (SNPs) previously described to be linked to diabetic kidney disease (DKD) in type 1 diabetes mellitus (T1DM). Additionally, we evaluated gene and protein expression related to the polymorphism associated with DKD.

**Methods:**

The association study included 1098 T2DM patients (718 with DKD and 380 without DKD). Out of the 13 polymorphisms associated with DKD in a previous study with T1DM, seven were chosen for evaluation in this sample: rs1888747, rs9521445, rs39075, rs451041, rs1041466, rs1411766 and rs6492208. The expression study included 91 patients who underwent nephrectomy. Gene expression was assessed by RT-qPCR and protein expression in kidney samples was quantified by western blot and it localization by immunohistochemistry.

**Results:**

The C/C genotype of rs1888747 SNP was associated with protection for DKD (OR = 0.6, 95 % CI 0.3–0.9; P = 0.022). None of the other SNPs were associated with DKD. rs1888747 is located near *FRMD3* gene. Therefore, FRMD3 gene and protein expression were evaluated in human kidney tissue according to rs1888747 genotypes. Gene and protein expression were similar in subjects homozygous for the C allele and in those carrying the G allele.

**Conclusions:**

Replication of the association between rs1888747 SNP and DKD in a different population suggests that this link is not the result of chance. rs1888747 SNP is located at the *FRMD3* gene, which is expressed in human kidney. Therefore, this gene is a candidate gene for DKD. However, in this study, no rs1888747 genotype or specific allele effect on gene and/or protein expression of the *FRMD3* gene was demonstrated.

**Electronic supplementary material:**

The online version of this article (doi:10.1186/s13098-015-0121-5) contains supplementary material, which is available to authorized users.

## Background

Diabetic kidney disease (DKD) is currently one of the main causes of end-stage renal disease (ESRD) [1–5], with an estimated incidence of 26 % in patients starting dialysis [6]. In addition to ESRD, DKD also causes an important increase in cardiovascular-related morbidity and mortality [6, 7].

An increasing body of evidence supports a genetic basis for DKD [8, 9]. However, little is known about the mode of transmission, which is probably polygenic, or about the mechanisms of gene-environment interaction. Until this moment, genome wide-association study (GWAS) and candidate gene studies have produced heterogeneous results [8–13].

A GWAS of 360,000 single nucleotide polymorphisms (SNPs) has identified 13 SNPs associated with DKD in two independent populations of patients with type 1 diabetes mellitus (T1DM) [14]. The strongest association was observed at the *FRMD3* (4.1 protein ezrin, radixin, moesin [FERM] domain containing 3) locus. Moreover, other relevant association was observed at *CARS* (cysteinyl-tRNA synthetase) locus. Confirmation of implicated SNPs was obtained in participants of the Diabetes Control and Complications Trial (DCCT)/Epidemiology of Diabetes Interventions and Complications (EDIC) study [15–17].

Because patients with T1DM and type 2 diabetes mellitus (T2DM) might share common genes related to DKD, the present study was designed to (1) investigate whether the SNPs found to be associated with DKD in T1DM are also associated with DKD in T2DM; (2) evaluate the association of these SNPs in early (microalbuminuria) and advanced stages of DKD (macroalbuminuria or ESRD); and (3) evaluate gene and protein expression of the genes associated with DKD in human kidney biopsies.

## Methods

### Patients analyzed in the association study

A case–control study was conducted with 1098 white T2DM patients selected from a cross-sectional study performed in the state of Rio Grande do Sul, Brazil [11]. T2DM was defined according to World Health Organization criteria [18]: diagnosis of diabetes after the age of 35 years, no use of insulin during the first year after diagnosis, and no episodes of diabetic ketoacidosis. Control T2DM patients were those with known diabetes duration of at least 5 years and normoalbuminuria (albuminuria <30 mg/24 h, n = 380). Cases were divided into two categories: early DKD (albuminuria 30–299 mg/24 h, n = 323) or advanced DKD (albuminuria ≥300 mg/24 h or ESRD, n = 395). The protocol was approved by the ethics committee, and all patients gave their written informed consent.

All patients underwent an evaluation that included a standardized questionnaire and physical examination, as previously described [19]. Briefly, information was collected about age, age at T2DM diagnosis, drug treatment and smoking. They were weighed bare feet, wearing light outdoor clothes and their height was measured. Body mass index (BMI) was calculated as weight (kg)/height^2^ (meters). For patients on dialysis, the mean of three weights measured after dialysis sessions were used. Hypertension was defined as blood pressure (BP) ≥140/90 mmHg or use of any antihypertensive medication. BP was defined based on the mean of two measurements in the sitting position using a standard mercury sphygmomanometer (phases I and V of Korotkoff). Diabetic retinopathy (DR) was assessed by fundus examination after mydriasis by an experienced ophthalmologist and graded as absent, non-proliferative or proliferative diabetic retinopathy.

Fasting blood samples were collected for laboratory and molecular analyses. Fasting plasma glucose was determined by a glucose oxidase method and HbA1c by an ion-exchange high performance liquid chromatography procedure (Merck-Hitachi L-9100 Glycated Hemoglobin Analyzer, Tokyo, Japan) with inter- and intra-assay coefficient of variation (CV) of 2.4 and 0.5 %, respectively (reference interval 4.1–6.0 %) [20]. Serum creatinine was determined by the Jaffé reaction [21]. Triglycerides and cholesterol levels were measured by enzymatic methods. LDL-cholesterol was calculated using the Friedewald equation. Urinary albumin excretion (UAE) was measured in 24 h-urine samples by immunoturbidimetry (Bayer, TarryTown, NY, USA), with intra- and interassay CV of 4.5 and 11 % respectively [22]. Use of angiotensin-converting enzyme inhibitors or angiotensin receptor antagonists was interrupted at least 1 week before albuminuria measurement.

### DNA isolation and genotyping

DNA was extracted from peripheral blood leukocytes by a standardized salting-out procedure. Seven out of 13 SNPs initially associated with DKD in T1DM in the GoKinD Study [15] were chosen to be included in the present study (rs1888747, rs9521445, rs39075, rs451041, rs1411766, rs6492208, rs1041466). Not all 13 SNPs were genotyped because some of them were in linkage disequilibrium (LD): rs1888747 and rs10868025 (*r*^2^ = 0.81), both located at chromosome 9q; rs39059 and rs39075 (7p, *r*^2^ > 0.96); rs451041 and rs739401 (11p, *r*^2^ > 0.87), rs9521445 and 7989848 (13q, *r*^2^ > 0.87), rs1411766 and 17412858 (13q, *r*^2^ = 1), and rs6492208 and rs2391777 (13q, *r*^2^ = 1) [15]. In LD pairs, the SNP having the strongest association with DKD was chosen for analysis. LD data was obtained from the HapMap Project (http://www.hapmap.org) for the Caucasian population. Polymorphism loci analyzed are shown in the supplementary material (Additional file 1: Table S1).

All polymorphisms were genotyped using primers and probes contained in the Human Custom TaqMan Genotyping Assay (40×) (Assays-By-Design Service, Life Technologies, Foster City, CA; USA). Primer and probe sequences used for genotyping these SNPs are described in Additional file 1: Table S2. Real-time (RT) PCR reactions were conducted in 96-well plates, in 5 µl total reaction volume using 2 ng of genomic DNA, TaqMan Genotyping Master Mix (1×) (Life Technologies), and Custom TaqMan Genotyping Assay (1×). Plates were positioned in a RT PCR thermal cycler (7500 Fast Real PCR System; Life Technologies) and heated for 10 min at 95 °C, followed by 40–50 cycles at 95 °C for 15 s and 60–62 °C for 1 min. The percentage of duplicates included in genotyping was 10 %. Genotyping success was more than 95 %, with a calculated error rate based on PCR duplicates of <0.01 %.

### FRMD3 expression in kidney tissue samples

For gene expression analysis, kidney tissue samples were collected from 91 patients undergoing therapeutic nephrectomy over a period of 30 months at Hospital de Clínicas de Porto Alegre. A standard questionnaire was used to collect information regarding age, gender, presence of arterial hypertension, diabetes mellitus, and smoking habit. A peripheral blood sample was collected from each subject for DNA extraction and genotyping. Excised normal kidney tissue was divided into aliquots for mRNA expression analyses and evaluation of protein expression and localization by western blot (WB) and immunohistochemistry (IHC), respectively. From 91 kidney samples analyzed for gene expression, only 48 kidney samples were utilized for protein expression analyzes, since 30 samples lacked enough material for proper protein extraction and the protein extraction was unsuccessful in 13 samples due to technical difficulties. The study protocol was approved by the Hospital’s ethics committees, and all patients gave their written informed consent.

### RNA isolation and quantification of *FRMD3* gene expression by real-time reverse transcription PCR

Kidney biopsies were homogenized in phenol/guanidine isothiocyanate (Invitrogen Life Technologies, Carlsbad, CA). RNA was extracted with chloroform and precipitated with isopropanol by centrifugation (12,000×*g*) at 4 °C. RNA pellet was washed twice with 75 % ethanol and resuspended in 10–50 μl of diethylpyrocarbonate treated water. Concentrations of isolated RNAs were assessed using NANODROP 2000 spectrophotometer (Thermo Scientific Inc., DE, USA). Only total RNA samples achieving adequate purity ratios (A260/A280 = 1.9–2.1) were used for subsequent analyses [23]. In addition, RNA integrity and purity were also checked on agarose gel containing Gel Red Nucleic Acid Gel Stain (Biotium Inc, Hayward, CA). The mean (± SD) concentration of isolated RNA was 2.68 ± 1.69 µg/250 mg kidney.

Real-time reverse transcription-PCR (RT-qPCR) was performed in two separate reactions: first, RNA was reverse transcribed into cDNA, and subsequently cDNA was amplified by RT-qPCR. Reverse transcription of 5 µg of RNA into cDNA was carried out using the Super Script Vilo Master Mix Kit (Synthesis System for RT-PCR; Invitrogen). RT-qPCR experiments were performed in a 7500 Real Time PCR System (Life Technologies). Experiments were performed by real-time monitoring of the increase in fluorescence of SYBER Green dye [24]. Primers were designed using Primer Express 3.0 Software (Life Technologies) and are depicted in Additional file 1: Table S2.

PCR reactions were performed using 10 µl of SYBER Green (1×) (Life Technologies), 1 µl of specific primers, *FRMD3* or *cyclophilin* A (Invitrogen), 7 µl of water and 1 μl of cDNA (0.2 μg/μl) in a total volume of 20 μl. Each sample was assayed in triplicate and a negative control was included in each experiment. The thermocycling conditions for these genes were as follows: an initial cycle of 95 °C for 10 min, followed by 50 cycles of 95 °C for 15 s and 60 °C for 1:30 min.

Quantification of *FRMD3* cDNA was performed using the ∆∆Cq method [23, 25] and expressed relative to the reference gene (*cyclophilin A*) in 91 kidney tissue samples: 76 carrying the G allele (GG or GC) of the rs1888747 SNP, and 15 with the CC genotype. Validation assays were done by separate amplification of the target (*FRMD3*) and reference (*cyclophilin A*) genes using serial dilutions of a cDNA sample. As a requirement of this method, both target and reference genes exhibited equal amplification efficiencies (E = 95–105 %) in all experiments. The ∆∆Cq method calculates changes in gene expression as relative fold differences (n-fold changes) between an experimental and an external calibrator sample [23]. RT-qPCR specificity was determined using melting curve analyses and all primers generated amplicons that produced a single sharp peak during the analyses [26].

### Determination of FRMD3 protein distribution and concentration in kidney

FRMD3 protein distribution and concentrations were determined in only 48 kidney sections, due to insufficient material for protein extraction. Thirty-nine of these sections belonged to subjects carrying the rs1888747 SNP G allele (GG = 23 and CG = 16). FRMD3 protein distribution was evaluated by IHC of formalin-fixed, paraffin-embedded kidney sections, while FRMD3 protein quantities were determined using WB. For IHC analyses, a goat rabbit anti-FRMD3 polyclonal antibody (Abcam, Cambridge, MA, USA) was used to detect FRMD3 protein distribution in human kidney tissue. IHC analyses were performed on 4 μm-thick kidney sections [27]. The routine IHC technique comprised: deparaffinization and rehydration, antigenic recovery, inactivation of endogenous peroxidase and blocking of nonspecific reactions. Slides were incubated with primary antibody and then incubated again with a biotinylated secondary antibody, streptavidin horseradish peroxidase conjugate (LSA; Dako Cytomation Inc, Carpinteria, CA, USA), and diamino benzidine tetra hydrochloride (Kit DAB Dako Cytomation Inc). Images were visualized through a Zeiss microscope (model AXIOSKOP-40; Carl Zeiss, Oberkochen, Germany) and captured using the Cool Snap-Pro CS (Media Cybernetics, Rockville, USA) camera.

For WB analyses, proteins from human kidneys were extracted using RIPA buffer and quantified using a colorimetric BCA™ Protein Assay Kit (Thermo Scientific). Protein extracts (20 µg) were resolved on 10 % polyacrylamide gels, transferred to Immobilon^®^-P^SQ^ membranes (Millipore, Billerica, MA, USA), and incubated with monoclonal antibodies to FRMD3 (Bioss, Woburn, MA, USA) or β-Actin (Millipore) overnight. Secondary antibodies consisted of horseradish peroxidase conjugated goat anti-rabbit antibodies (Millipore). Detection was performed using Immobilon Western Chemiluminescent HRP Substrate (Millipore), and images were acquired and quantified in an Image Quant LAS 500 (GE Healthcare, Pittsburgh, USA) digital imaging system. Initially, the process was applied for FRMD3 antibody. After stripping, the membrane was re-blotted with β-Actin antibody. Data are expressed as arbitrary units (AU). Three isoforms of FRMD3 protein (http://www.uniprot.org/uniprot/A2A2Y4) were quantified separately (data not shown) and grouped by WB.

### Statistical analyses

In the association study, we compared controls vs. cases (defined as any degree of DKD) and controls vs. different DKD stages (microalbuminuria, macroalbuminuria/ESRD). Data were analyzed assuming dominant, recessive and additive inheritance models to determine the best fit. Allele frequencies of all SNPs were determined by gene counting, and departures from Hardy-Weinberg equilibrium (HWE) were verified using the Chi square (χ^2^) test. Allele and genotype frequencies were compared between groups using the χ^2^ test. Continuous data with normal distribution are presented as mean ± SD, while continuous variables with skewed distribution were log-transformed before analysis and are presented as medians (minimum–maximum values), with exception of FRMD3 protein concentration (WB) for which log-transformation did not normalize the variable, thus we used the non-log variable and non-parametric tests. Categorical data are expressed as number of cases and percent of individuals affected. One-way analysis of variance (ANOVA), χ^2^ or Student’s t test were used to compare the groups in terms of clinical and laboratory characteristics or FRMD3 gene/protein expression. The magnitude of the association was estimated using odds ratios (ORs) with 95 % confidence interval (95 % CI). Multiple logistic regression analysis was used to evaluate the independence of possible SNP associations with DKD, adjusting for covariables. Pearson’s correlation test was used to evaluate correlation between quantitative variables. Multiple linear regression analyses were performed with FRMD3 gene or protein expression as dependent variables and age and sex as independent variables. Results for which P < 0.05 were considered statistically significant. All statistical analyses were performed using SPSS 18.0 (Chicago, IL, USA).

## Results

### Association study

We evaluated 1098 T2DM patients, of which 718 (65.4 %) had DKD (323 microalbuminuric and 395 macroalbuminuric/ESRD subjects). Patients with DKD had higher BP, higher prevalence of DR, and a worse lipid profile compared to the control group (Additional file 1: Table S3). Both groups had similar HbA1c levels.

Genotypes of the SNPs analyzed in the present study were in HWE (P > 0.05), with exception of the rs1411766 and rs6492208 SNPs. Because no genotyping errors were detected, these SNPs were maintained in the study. Genotype distributions are shown in Table 1. Of these seven SNPs, only rs1888747 was significantly associated with DKD (CC/CG/GG = cases vs. controls = 6.8/41.5/51.7 % vs 10.8/40.0/49.2 %, P = 0.037). Minor allele frequencies were 0.31 and 0.27 in controls and cases (microalbuminuric + macroalbuminuric/ESRD), respectively (P = 0.06). The strongest association was observed assuming recessive (CC vs. CG/GG, OR: 0.60, 95 % CI 0.39–0.94; P = 0.031) and additive (CC vs. GG, OR: 0.60, 95 % CI 0.38–0.95; P = 0.036) models. This association persisted after controlling for T2DM duration, gender, systolic BP, and triglycerides (OR: 0.52, 95 % CI 0.39–0.90; P = 0.021). In contrast with the study by Pezolessi et al. [17], the rs451041 polymorphism in the *CARS* gene was not associated with DKD in this sample of T2DM patients. Moreover, none of the other analyzed SNPs was associated with this disease (Table 1).

**Table 1:** Genotype distributions in T2DM patients with (cases) or without DKD (controls)

Polymorphism	Controls (n = 380)	Cases (n = 718)	P	Dominant: OR (95 % CI)/P	Recessive: OR (95 % CI)/P	Additive: OR (95 % CI)/P
rs1888747						
CC	10.8 (41)	6.8 (49)	0.037	0.91 (0.71–1.16)/0.476	0.60 (0.39–0.94)/0.031	0.60 (0.38–0.95)/0.036
CG	40.0 (152)	41.5 (298)				
GG	49.2 (187)	51.7 (371)				
rs9521445						
AA	18.9 (72)	22.3 (160)	0.146	0.94 (0.71–1.23)/0.688	1.23 (0.90–1.67)/0.226	1.12 (0.78–1.61)/0.598
AC	53.2 (202)	48.5 (348)				
CC	27.9 (106)	29.2 (210)				
rs39075						
AA	17.9 (68)	19.4 (139)	0.139	0.85 (0.66–1.11)/0.264	1.10 (0.80–1.52)/0.611	0.97 (0.68–1.40)/0.963
AG	49.5 (188)	44.4 (319)				
GG	32.6 (124)	36.2 (260)				
rs451041						
AA	23.4 (89)	21.3 (153)	0.318	0.86 (0.65–1.13)/0.305	0.89 (0.66–1.19)/0.467	0.82 (0.58–1.16)/0.296
AG	48.2 (183)	47.1 (338)				
GG	28.4 (108)	31.6 (227)				
rs1041466						
GG	14.2 (54)	17.3 (124)	0.059	0.87 (0.67–1.13)/0.314	1.26 (0.89–1.78)/0.221	1.11 (0.76–1.63)/0.659
AG	51.1 (194)	44.7 (321)				
AA	34.7 (132)	38.0 (273)				
rs1411766						
AA	10.0 (38)	12.6 (91)	0.399	1.25 (0.97–1.60)/0.095	1.31 (0.88–1.95)/0.226	1.41 (0.93–2.13)/0.131
AG	34.0 (129)	36.8 (264)				
GG	56.0 (213)	50.6 (363)				
rs6492208						
CC	20.7 (79)	19.9 (143)	0.461	1.01 (0.78–1.31)/1.000	0.95 (0.70–1.29)/0.792	0.96 (0.68–1.36)/0.898
CT	43.9 (167)	45.0 (323)				
TT	35.4 (134)	35.1 (252)				

Data are presented as % (n)

*T2DM* type 2 diabetes mellitus, *DKD* diabetic kidney disease

Table 2 shows the genotype distribution of rs1888747 SNP according to renal status (normo-, micro- or macro/ESRD). The same pattern described above was observed, with a decrease in the frequency of the homozygous genotype (CC) for the minor allele of the rs1888747 SNP in subjects with microalbuminuria or macroalbuminuria/ESRD compared to controls. The frequency of the C allele was also lower in individuals with microalbuminuria or macroalbuminuria/ESRD compared to normoalbuminuric controls. In addition, genotype distribution was compared between normo- and macro/ESRD groups (Table 2), and the same pattern was observed.

**Table 2:** Genotype distribution of rs1888747 polymorphism according to renal status

Renal status					
Genotype	Normoalbuminuria (N = 380)	Microalbuminuria (N = 323)	Macro/ESRD (N = 395)	P*	P**
CC	41 (10.8)	25 (7.7)	24 (6.3)	0.04	0.05
GC	152 (40.0)	138 (42.7)	160 (40.9)		
GG	187 (49.2)	160 (49.5)	211 (52.8)		
C allele	0.31	0.29	0.26	0.02	0.03

Data expressed as number of cases (%)

* P for comparisons among the three groups (normo- vs. micro- vs. macroalbuminuric/ESRD)

** P for comparison between normo- and macro/ESRD groups

### FRMD3 expression study: sample description

Since only rs1888747 was associated with DKD in this sample of subjects with T2DM, and this SNP is located near *FRMD3* gene promoter region, we evaluated FRMD3 gene and protein expression in human kidney tissue. The mean age of the 91 subjects for the rs1888747 SNP was 57.7 ± 14.2 years; 47.8 % were males, 55.4 % had arterial hypertension, 23.9 % were smokers, and 19.6 % had diabetes. Additional file 1: Table S4 presents the clinical data according to rs1888747 genotypes. Genotype frequencies were as follows: GG, 51.1 % (n = 47), GC, 31.5 % (n = 29), and CC, 16.3 % (n = 15). The minor allele (C) frequency was 32 % in this sub-sample.

### *FRMD3* mRNA expression

**Fig. 1:**
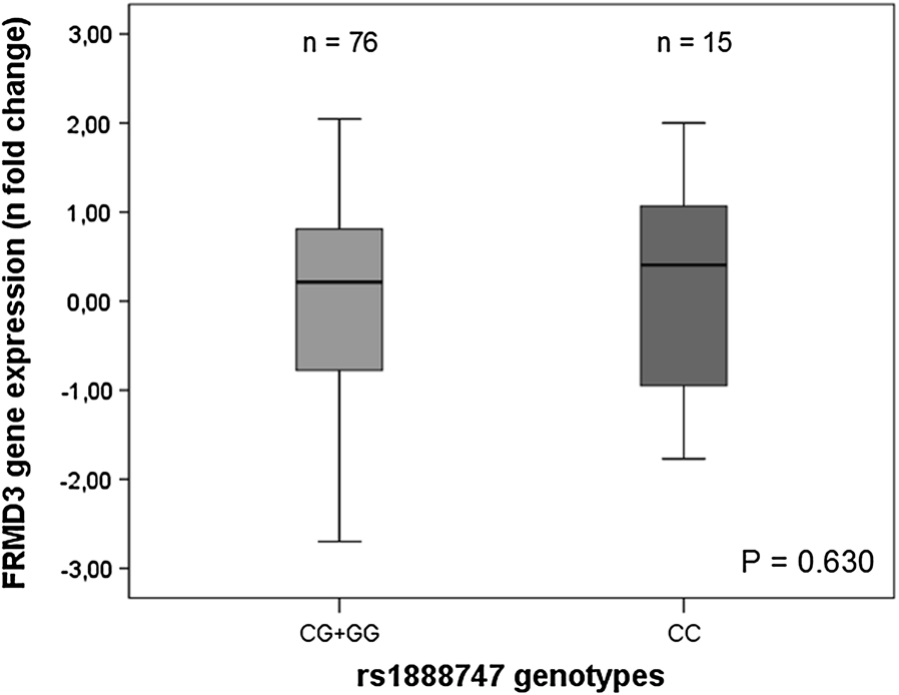
*FRMD3* gene expression in kidney samples according to the *FRMD3* rs1888747 polymorphism (recessive model). P value was computed using Student’s *t* test. Results are expressed as n-fold differences from the calibrator sample and are presented as medians (minimum–maximum values)

*FRMD3* mRNA expression in kidney tissue samples categorized according to the minor allele recessive model (CC vs. GG + GC) for the rs1888747 polymorphism are depicted in Fig. 1. Gene expression was similar in subjects homozygous for the C allele and in those carrying the G allele [0.41 (−1.77 to 2.00) vs. 0.21 (−2.70 to 2.05), respectively; P = 0.630]. *FRMD3* mRNA expression did not correlate with age (r^2^ = 0.11; P = 0.305), and did not differ between females and males [0.31 (−1.92 to 2.05) vs. 0.12 (−2.70 to 2.00); P = 0.200], between patients with or without hypertension [0.47 (−2.05 to 2.05) vs. −0.21 (−2.70 to 1.99); P = 0.077] and between patients with or without diabetes [0.37 (−2.70 to 1.98) vs. 0.16 (−2.22 to 2.05); P = 0.779].

It is worth noting that when separating diabetic from non-diabetic subjects, *FRMD3* mRNA expression was also similar between genotype groups: CC (n = 4) vs. GG + CG (n = 25) [0.34 (−2.70 to 1.98) vs. 0.54 (0.13–1.17); P = 0.306] for the diabetic group, and CC (n = 9) vs. GG + CG (n = 48) [0.18 (−2.22 to 2.05) vs. −0.43 (−1.77 to 2.00); P = 0.900] for the non-diabetic group.

### FRMD3 protein expression

**Fig. 2:**
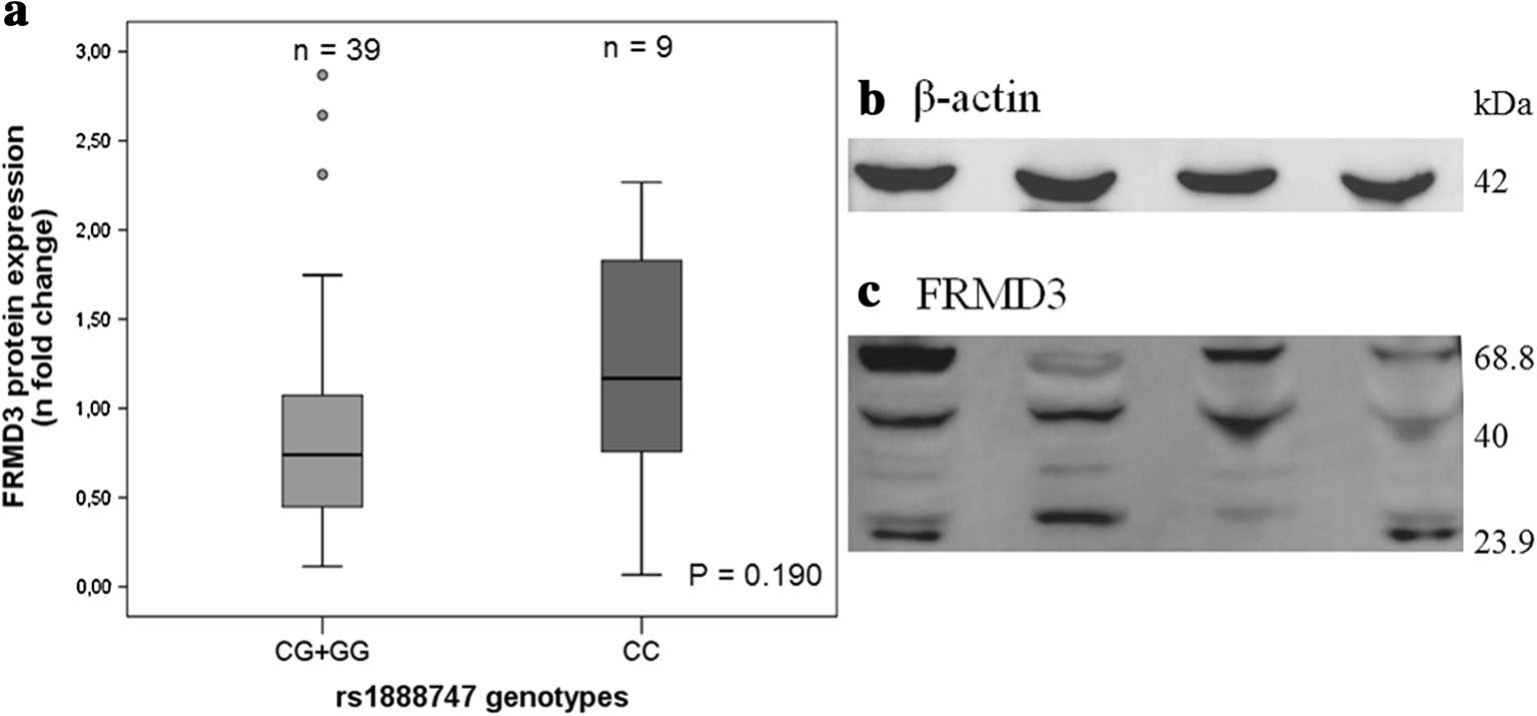
FRMD3 protein expression according to the *FRMD3* rs1888747 polymorphism (recessive model). **a** FRMD3 protein expression according to the *FRMD3* rs1888747 polymorphism (recessive model). P value was computed using Student’s* t* test. Results are expressed as n-fold changes and are presented as medians (minimum–maximum values). Representative western blots for: **b** β-actin, and **c** three isoforms of FRMD3

FRMD3 protein expression in kidney tissue samples categorized according to the recessive model (CC vs. GG + GC) of the rs1888747 polymorphism is shown in Fig. 2. FRMD3 total protein expression was similar in subjects homozygous for the C allele and in those carrying the G allele [1.17 (0.07–2.27) vs. 0.74 (0.11–2.87), respectively; P = 0.190). Moreover, *FRMD3* rs1888747 genotypes did not influence FRMD3 protein levels after adjustment for age, sex and presence of DM (β = −0.019, P = 0.908). However, FRMD3 protein concentrations negatively correlate with *FRMD3* gene expression (r^2^ = −0.375; P = 0.013). It is worth mentioning that when FRMD3 isoforms were quantified separately (data not shown), the data were similar to the quantification of the grouped isoforms.

**Fig. 3:**
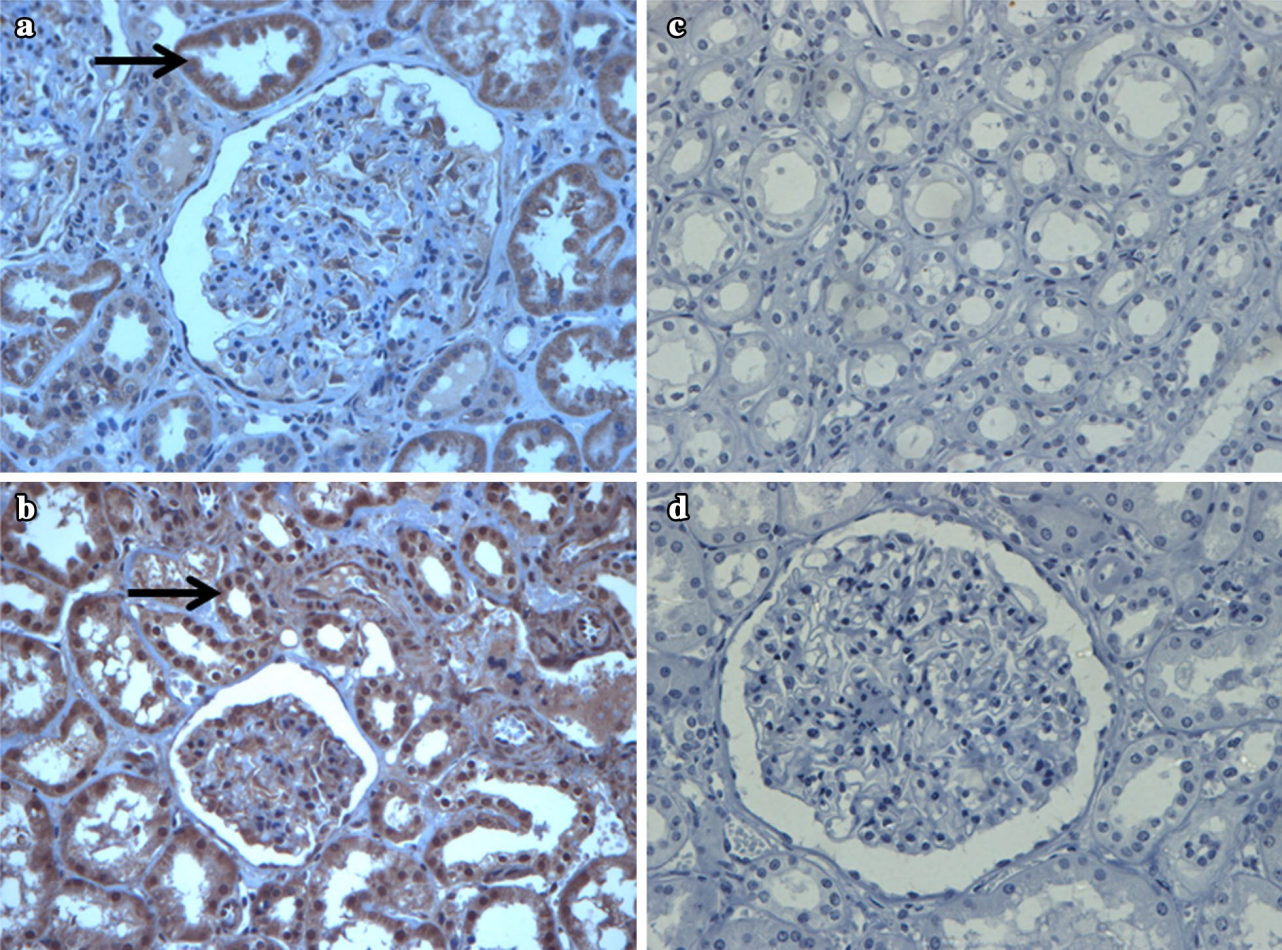
Representative photomicrographs of human kidney by immunohistochemistry for FRMD3 protein. **a**, **b** FRMD3 protein expression is predominantly localized in tubules. In glomerus, the expression is localized exclusively in podocytes. *Arrows* indicate different areas of FRMD3 immunostaining. **c**, **d** Negative control samples. Original magnification of all images ×400

Figure 2 depicts representative WB gels for FRMD3 protein. Representative IHC photomicrographs of human kidney are represented in Fig. 3. FRMD3 protein was predominantly observed in tubules and podocytes. FRMD3 protein expression did not correlate with age (r^2^ = −0.13; P = 0.364) and was similar in females and males [0.73 (0.11–2.87) vs. 0.79 (0.07–2.64); P = 0.959] and in patients with or without arterial hypertension [0.73 (0.07–2.31) vs. 1.05 (0.11–2.87); P = 0.212]. FRMD3 protein expression reached borderline formal statistical significance when comparing patients with or without diabetes [0.66 (0.11–2.31) vs. 0.99 (0.07–2.87); P = 0.05].

When separating diabetic from non-diabetic subjects, FRMD3 protein levels appeared to be similar between groups: CC (n = 2) vs. GG + CG (n = 20) [1.27 (0.27–2.27) vs. 0.66 (0.16–2.31); P = 0.193] for the diabetic group, and CC (n = 5) vs. GG + CG (n = 18) [1.17 (0.07–1.85) vs. 0.90 (0.12–2.87); P = 0.969] for the non-diabetic group. Nevertheless, this result should be cautiously interpreted due to limited statistical power.

## Discussion

In the present study, we found that rs1888747 SNP is associated with DKD in T2DM, as previously described for T1DM patients [15]. The CC genotype conferred protection against the development of DKD. Additionally to previous data from T1DM [15], a subgroup with microalbuminuria was evaluated. The confirmation of this SNP with DKD in our population should be considered carefully, since frequently genetic association studies failed to be replicate in additional populations [13, 28, 29]. DKD is probably a multifactorial disorder resulting from an interaction between environmental and genetic factors. GWAS are capable of identifying unknown chromosomal regions that may be involved in the pathogenesis of DKD. However, replication of the findings in distinct populations is essential to confirm the associations observed in such studies.

The rs1888747 SNP (C/G) is an intergenic polymorphism, located near the promoter region of the *FRMD3* gene [30]. This gene encodes a structural/cytoskeletal protein involved in maintaining cellular shape [31, 32], but whose function remains otherwise unknown. Studies focusing on the relation between rs1888747 and DKD have been performed in T1DM and T2DM individuals of various ethnic backgrounds, such as Caucasians, Japanese, African Americans, and Chinese, with conflicting results [15, 33–37].

A second SNP found in the GoKinD sample collection and replicated in DCCT/EDIC subjects is the rs451041 SNP, located in chromosome 11p. The nearest gene to rs451041 is *CARS*, which is expressed in mesangial and proximal tubule cells. Mutations in this gene have been associated with neurodegenerative diseases and cystinosis [38, 39]. However, neither this nor the other SNPs originally associated with DKD in T1DM patients were associated with DKD in the present study with patients with T2DM. Although this finding requires further validation, if confirmed it might suggest that different genes are involved in the development of DKD in T1DM and T2DM.

The evidence to infer susceptibility genes contributing to the pathogenesis of DKD remains elusive. Advances in genotyping technologies have allowed the identification of several chromosomal regions potentially associated with DKD. Nevertheless, methodological limitations such as lack of statistical power, differences regarding the population studied and phenotypes analyzed make it difficult to compare some of the study findings and build up on results.

In the present study, we also aimed to evaluate if the rs1888747 risk allele was influencing FRMD3 gene and/or protein expression. Unfortunately, we were not able to demonstrate an association of these variables with the different genotypes. The *FRMD3* gene (ID: 257019) is located on chromosome 9q21.32, size of 2.282 pb; 21 exons; 81.4 Mb position (http://www.ncbi.nlm.nih.gov/gene/257019). Members of the protein 4.1 family have well characterized functions in a variety of cell types, including rat nephron [31, 32]. *FRMD3* is present in adult ovaries as well as fetal skeletal muscle, brain, and thymus [40]. Our findings confirm that FRMD3 is expressed in proximal renal tubular cells and human podocytes, as previously shown [14, 35], suggesting that this protein is involved in maintaining the function and integrity of the slit diaphragm. Therefore, it is a good candidate gene for albuminuria development. Furthermore, FRMD3 protein concentrations negatively correlated with *FRMD3* gene expression. Although this needs further confirmation, it suggests that *FRMD3* mRNA levels may not reflect the expression of the protein itself, probably due to important translational regulation mechanisms.

Recently, changes in *FRMD3* expression have been linked to progression of DKD in a group of 22 Pima Indians with T2DM [41]. The authors proposed an influence of the rs1888747 polymorphism in the *FRMD3* promoter on transcriptional regulation within the bone morphogenetic protein (BMP)-signaling pathway [41], suggesting that the transcriptional coregulation of BMP pathway members and *FRMD3* might be mediated by the four transcription factor binding site (TFBS) promoter modules in the functional context of DKD [42]. The mechanism mediating the connection between *FRMD3* and BMP pathway members remains unknown. No evidence at the protein, RNA, or microRNA levels was found [42]. The present findings, derived from genotyping, gene and protein expression studies in human kidney tissue, are important to elucidate the role of the rs1888747 polymorphism. Even so, further research is necessary to confirm our observations and to unveil the mechanisms underlying the association of the rs1888747 polymorphism with DKD.

A limitation of case–control studies of DKD such as the present one is survival bias. However, we do not believe that this had an impact on the results we report, because minor allele frequency was similar in mild and severe stages of DKD, although data from the prospective DCCT/EDIC cohort [16] suggest that the rs1888747 G allele had a faster progression to severe DKD. Another possible limitation of our study is the fact that the samples for the expression studies were not obtained from patients with DKD, but instead from patients undergoing therapeutic nephrectomy. Thus, the present gene and protein expression findings cannot be directly translated to patients with DKD. Further studies are necessary to determine if the rs1888747 SNP is or not related to changes in FRMD3 gene and protein expression in DKD.

In conclusion, the present study replicates the protective effect of rs1888747 SNP against established DKD previously described for T1DM and T2DM. Moreover, in agreement with previous data, it also shows that this effect is already present in less advanced stages of DKD, such as microalbuminuria. Outside the context of diabetes, this study was not able to show an influence of rs1888747 SNP on FRMD3 gene and/or protein expression.

## Additional file

10.1186/s13098-015-0121-5 Polymorphisms analyzed. **Table S2.** Primer sequences used to genotype polymorphisms and to analyze gene expression. **Table S3.** Clinical characteristics of the subjects included in the association study. **Table S4.** Clinical characteristics of subjects included in the expression study according to rs1888747 polymorphism.

## Abbreviations

BMI: body mass index
BP: blood pressure
CARS: cysteinyl-tRNAsynthetase
DCCT/EDIC: diabetes control and complications trial/Epidemiology of diabetes interventions and complications
DKD: diabetic kidney disease
DR: diabetic retinopathy
ESRD: end-stage renal disease
FRMD3: 4.1 protein ezrin, radixin, moesin [FERM] domain containing 3
GWAS: genome wide-association study
HWE: Hardy–Weinberg equilibrium
IHC: immunohistochemistry
LD: linkage disequilibrium
RT-qPCR: quantitative real-time PCR
SNPs: Single nucleotide polymorphisms
T1DM: type 1 diabetes mellitus
T2DM: type 2 diabetes mellitus
UAE: urinary albumin excretion
WB: Western blot

## References

[1] Foley RN, Collins AJ (2007). End-stage renal disease in the United States: an update from the United States Renal Data System. J Am Soc Nephrol.

[2] Bloomgarden ZT (2008). Diabetic nephropathy. Diabetes Care.

[3] Prevention., C.f.D.C.a., National diabetes fact sheet: national estimates and general information on diabetes and prediabetes in the United States. 2011, Atlanta, GA: US Department of Health and Human Services.

[4] Tapp RJ (2004). Albuminuria is evident in the early stages of diabetes onset: results from the Australian Diabetes, Obesity, and Lifestyle Study (AusDiab). Am J Kidney Dis.

[5] Lim A (2014). Diabetic nephropathy—complications and treatment. Int J Nephrol Renovasc Dis.

[6] Bruno RM, Gross JL (2000). Prognostic factors in Brazilian diabetic patients starting dialysis: a 3.6-year follow-up study. J Diabetes Complications.

[7] Valmadrid CT (2000). The risk of cardiovascular disease mortality associated with microalbuminuria and gross proteinuria in persons with older-onset diabetes mellitus. Arch Intern Med.

[8] Krolewski AS (2006). A genome-wide linkage scan for genes controlling variation in urinary albumin excretion in type II diabetes. Kidney Int.

[9] Vardarli I (2002). Gene for susceptibility to diabetic nephropathy in type 2 diabetes maps to 18q22.3-23. Kidney Int.

[10] Leitao CB (2008). The role of K121Q ENPP1 polymorphism in diabetes mellitus and its complications. Braz J Med Biol Res.

[11] Canani LH (2005). The fatty acid-binding protein-2 A54T polymorphism is associated with renal disease in patients with type 2 diabetes. Diabetes.

[12] Murea M (2011). Genome-wide association scan for survival on dialysis in African-Americans with type 2 diabetes. Am J Nephrol.

[13] Thameem F (2013). A genome-wide search for linkage of estimated glomerular filtration rate (eGFR) in the Family Investigation of Nephropathy and Diabetes (FIND). PLoS One.

[14] Mueller PW (2006). Genetics of Kidneys in Diabetes (GoKinD) study: a genetics collection available for identifying genetic susceptibility factors for diabetic nephropathy in type 1 diabetes. J Am Soc Nephrol.

[15] Pezzolesi MG (2009). Genome-wide association scan for diabetic nephropathy susceptibility genes in type 1 diabetes. Diabetes.

[16] Nathan DM (2005). Intensive diabetes treatment and cardiovascular disease in patients with type 1 diabetes. N Engl J Med.

[17] The Diabetes Control and Complications Trial Research Group (DCCT) (1993). The effect of intensive treatment of diabetes on the development and progression of long-term complications in insulin-dependent diabetes mellitus. N Engl J Med.

[18] Report of the expert committee on the diagnosis and classification of diabetes mellitus. Diabetes Care. 2003; 26(Suppl 1): S5–20. 10.2337/diacare.26.2007.s5 12502614

[19] Canani LH, Gerchman F, Gross JL (1998). Increased familial history of arterial hypertension, coronary heart disease, and renal disease in Brazilian type 2 diabetic patients with diabetic nephropathy. Diabetes Care.

[20] Camargo JL, Felisberto M, Gross JL (2004). Effect of pre-analytical variables on glycohemoglobin measurements in routine clinical care. Clin Biochem.

[21] Junge W (2004). Determination of reference intervals for serum creatinine, creatinine excretion and creatinine clearance with an enzymatic and a modified Jaffe method. Clin Chim Acta.

[22] Zelmanovitz T (1997). The receiver operating characteristics curve in the evaluation of a random urine specimen as a screening test for diabetic nephropathy. Diabetes Care.

[23] Bustin SA (2009). The MIQE guidelines: minimum information for publication of quantitative real-time PCR experiments. Clin Chem.

[24] Higuchi R (1993). Kinetic PCR analysis: real-time monitoring of DNA amplification reactions. Biotechnology (N Y).

[25] Livak KJ, Schmittgen TD (2001). Analysis of relative gene expression data using real-time quantitative PCR and the 2(−Delta Delta C(T)) Method. Methods.

[26] Brondani LA (2012). The UCP1-3826A/G polymorphism is associated with diabetic retinopathy and increased UCP1 and MnSOD2 gene expression in human retina. Invest Ophthalmol Vis Sci.

[27] Hsu SM, Raine L, Fanger H (1981). The use of antiavidin antibody and avidin-biotin-peroxidase complex in immunoperoxidase technics. Am J Clin Pathol.

[28] Palmer ND (2014). Evaluation of candidate nephropathy susceptibility genes in a genome-wide association study of African American diabetic kidney disease. PLoS One.

[29] McKnight AJ, McKay GJ, Maxwell AP (2014). Genetic and epigenetic risk factors for diabetic kidney disease. Adv Chronic Kidney Dis.

[30] Palmer ND, Freedman BI (2013). Diabetic nephropathy: FRMD3 in diabetic nephropathy–guilt by association. Nat Rev Nephrol.

[31] Hoover KB, Bryant PJ (2000). The genetics of the protein 4.1 family: organizers of the membrane and cytoskeleton. Curr Opin Cell Biol.

[32] Ramez M (2003). Distinct distribution of specific members of protein 4.1 gene family in the mouse nephron. Kidney Int.

[33] Mooyaart AL (2011). Genetic associations in diabetic nephropathy: a meta-analysis. Diabetologia.

[34] Freedman BI (2011). Differential effects of MYH9 and APOL1 risk variants on FRMD3 Association with Diabetic ESRD in African Americans. PLoS Genet.

[35] Pezzolesi MG (2013). Family-based association analysis confirms the role of the chromosome 9q21.32 locus in the susceptibility of diabetic nephropathy. PLoS One.

[36] Maeda S (2010). Replication study for the association between four Loci identified by a genome-wide association study on European American subjects with type 1 diabetes and susceptibility to diabetic nephropathy in Japanese subjects with type 2 diabetes. Diabetes.

[37] Williams WW (2012). Association testing of previously reported variants in a large case-control meta-analysis of diabetic nephropathy. Diabetes.

[38] Antonellis A, Green ED (2008). The role of aminoacyl-tRNA synthetases in genetic diseases. Annu Rev Genomics Hum Genet.

[39] Gahl WA, Thoene JG, Schneider JA (2002). Cystinosis. N Engl J Med.

[40] Ni X (2003). Molecular cloning and characterization of the protein 4.1O gene, a novel member of the protein 4.1 family with focal expression in ovary. J Hum Genet.

[41] Martini S (2013). From single nucleotide polymorphism to transcriptional mechanism: a model for FRMD3 in diabetic nephropathy. Diabetes.

[42] Haase D (2007). FRMD3, a novel putative tumour suppressor in NSCLC. Oncogene.

