# Supplementary material for: rs1888747 polymorphism in the FRMD3 gene, gene and protein expression: role in diabetic kidney disease
Source: Diabetol Metab Syndr. 2016 Jan 8;8:3. doi: 10.1186/s13098-015-0121-5 (PMC4706705; doi:10.1186/s13098-015-0121-5)
Supplement: Supplementary file 1 — 10.1186/s13098-015-0121-5 Polymorphisms analyzed. Table S2. Primer sequences used to genotype polymorphisms and to analyze gene expression. Table S3. Clinical characteristics of the subjects included in the association study. Table S4. Clinical characteristics of subjects included in the expression study according to rs1888747 polymorphism. [file 13098_2015_121_MOESM1_ESM.docx]

**Table S1 - Polymorphisms analyzed**

| **Polymorphism** | **Alelles** | **Chromossome** | **Near gene** |
| --- | --- | --- | --- |
| **rs39075** | A/G | 7p | CHN2 |
| **rs1888747** | C/G | 9q | FRMD3 |
| **rs451041** | G/A | 11q | CARS |
| **rs9521445** | C/A | 13q | __ |
| **rs1041466** | A/G | 13q | __ |
| **rs1411766** | G/A | 13q | __ |
| **rs6492208** | C/T | 13q | __ |

**Table S2 - Primer sequences used to genotype polymorphisms and to analyze gene expression**

| rs39075 | F 5' - CCCAAGGTGCCAAGGATGA - 3' |
| --- | --- |
|  | R 5' - GCTGTTAGCAAAGAAAGCACTTCA - 3' |
|  | VIC 5' - CAGTACCGAAACAGC - 3' |
|  | FAM 5' - CAGTACCAAAACAGC - 3' |
| rs451041 | F 5' - CTCCCACCTGCCAAGTAGTG - 3' |
|  | R 5' - GGCTCATTTCATTAAGCGTCAGAA - 3' |
|  | VIC 5' - AAGGCCTAACATGATC - 3' |
|  | FAM 5' - AAGGCCTAACGTGATC - 3' |
| rs9521445 | F 5' - CCTTACTCTCTAGCCCCAAGTTGA - 3' |
|  | R 5' - GACTATTCAGACGAATCTGGACACA - 3' |
|  | VIC 5' - TGGAAGTGCAAGGTTA - 3' |
|  | FAM 5' - CTTGGAAGTTCAAGGTTA - 3' |
| rs1041466 | F 5' - GCATTTTTATTGACTGGGACAGCTT - 3' |
|  | R 5' - CTCAGTGACTACAGAAATTACCAGGAA - 3' |
|  | VIC 5' - AAGCACTGTCATTACC - 3' |
|  | FAM 5' - AAGCACTGTCGTTACC - 3' |
| rs1411766 | F 5' - CACTTCTCTTCCTCTGTCTCCAAAA - 3' |
|  | R 5' – CCTAGGAAAAGGCGGCTAGTTG - 3' |
|  | VIC 5' - CATTCGTGCGATTCAG - 3' |
|  | FAM 5' - TCATTCGTGTGATTCAG - 3' |
| rs6492208 | F 5' - CCACACCATGCCAACTACTTCAT - 3' |
|  | R 5' - CCGACTAAATCAGTGTTCATGACAAC - 3' |
|  | VIC 5' - TGTTGATAATGCACTTGACAAT - 3' |
|  | FAM 5' - TTGATAATGCACCTGACAAT - 3' |
| rs1888747 | F 5' - TCACCTGGATTGAACTACCCAATG - 3' |
|  | R 5' - GAGTTCATTCTTTGAGTTCTTCCTAATCTTCATA - 3' |
|  | VIC 5' - CAATGGCCAGTTGGTTT - 3' |
|  | FAM 5' - AATGGCCACTTGGTTT - 3' |
| FRMD3 gene* | F 5' - TTTTCCCCAAGCAGTCACA- 3' |
|  | R 5' - TGCCCCCTGAGTTCATTTT- 3' |
| Cyclophilin gene* | F 5' - GCCGATGACGACCCTTG- 3' |
|  | R 5' - TGCCGCCAGTGCCATTATG- 3' |

F, forward primer; R, reverse primer.

* Primers were designed using published human gene sequences and Primer Express 3.0 software (Life Technologies) and projected to target two consecutive exons, so as to prevent the amplification of any contaminating genomic DNA.

**Table S3 - Clinical characteristics of the subjects included in the association study**

|  | **Controls** | **Cases** | **P** |
| --- | --- | --- | --- |
|  | **n = 380** | **n = 718** |  |
| **Age (years)** | 60.4 ± 9.7 | 59.4 ± 10.8 | 0.131 |
| **Male - n (%)** | 142 (37.4) | 424 (59.0) | <0.001 |
| **Duration of diabetes (years)** | 14.2 ± 7.4 | 14.9 ± 10.0 | 0.230 |
| **Body mass index (kg/m2)** | 29.1 ± 5.3 | 28.8 ± 5.3 | 0.372 |
| **SBP (mmHg)** | 140.8 ± 22.7 | 145.1 ± 23.5 | 0.004 |
| **DBP (mmHg)** | 84.9 ± 12.6 | 85.3 ± 12.2 | 0.351 |
| **Diabetic retinopathy - n (%)** | 125 (32.9) | 607 (84.5) | <0.001 |
| **Smoking n - (%)** | 65 (17.1) | 118 (16.4) | 0.841 |
| **FPG (mg/dl)** | 168.7 ± 63.0 | 178.0 ± 82.5 | 0.055 |
| **HbA1c (%)** | 7.31 ± 1.93 | 7.35 ± 2.12 | 0.759 |
| **Total cholesterol (mg/dl)** | 207.7 ± 43.2 | 201.3 ± 50.2 | 0.035 |
| **HDL cholesterol (mg/dl)** | 46.1 ± 11.5 | 43.1 ± 12.4 | <0.001 |
| **Triglycerides (mg/dl)** | 143 (26-946) | 175 (46 -1669) | <0.001 |
| **Serum creatinine (mg/dl)** | 0.89 ± 0.20 | 2.67 ± 2.94 | <0.001 |

Data are mean ± SD, median (range) or number of cases (%). Where: SBP = systolic blood pressure; DBP = diastolic blood pressure; FPG = fasting plasma glucose.

**Table S4 - Clinical characteristics of subjects included in the expression study according to rs1888747 polymorphism**

|  | **C/C** | **C/G** | **G/G** | **P** |
| --- | --- | --- | --- | --- |
| **Gene expression study** | **n=15** | **n=29** | **n=47** |  |
| Age (years) | 50.4 ± 18.4 | 58.9 ± 12.2 | 58.9 ± 13.2 | 0.102 |
| Male Gender (%) | 5 (33,3) | 14 (48.3) | 24 (51.1) | 0.484 |
| Hypertension (%) | 9 (69.2) | 15 (62.5) | 26 (61.9) | 0.888 |
| Body Mass Index (%) | 28.2 ± 4.2 | 29.8 ± 6.7 | 28.9 ± 6.1 | 0.734 |
| Diabetes (%) | 4 (30.8) | 6 (21.4) | 19 (42.2) | 0.183 |
| **Protein expression study** | **n=9** | **n=16** | **n=23** |  |
| Age (years) | 59.4 ± 15.3 | 57.2 ± 11.3 | 63.8 ± 12.8 | 0.290 |
| Male Gender (%) | 4 (44.4) | 7 (43.8) | 12 (52.2) | 0.851 |
| Hypertension (%) | 6 (75.0) | 9 (69.2) | 14 (66.7) | 0.910 |
| Body Mass Index (%) | 28.2 ± 4.55 | 29.36 ± 6.41 | 28.1 ± 5.5 | 0.845 |
| Diabetes (%) | 2 (28.6) | 5 (33.3) | 15 (65.2) | 0.080 |

Data expressed as mean ± SD or number of cases (%).
